# Results from an Audit Feedback Strategy for Chronic Obstructive Pulmonary Disease In-Hospital Care: A Joint Analysis from the AUDIPOC and European COPD Audit Studies

**DOI:** 10.1371/journal.pone.0110394

**Published:** 2014-10-15

**Authors:** Jose Luis Lopez-Campos, M. Isabel Asensio-Cruz, Ady Castro-Acosta, Carmen Calero, Francisco Pozo-Rodriguez, on behalf of the AUDIPOC and the European COPD Audit studies

**Affiliations:** 1 Unidad Médico-Quirúrgica de Enfermedades Respiratorias, Instituto de Biomedicina de Sevilla (IBiS), Hospital Universitario Virgen del Rocio/Universidad de Sevilla, Sevilla, Spain; 2 Centro de Investigación Biomédica en Red de Enfermedades Respiratorias (CIBERES), Instituto de Salud Carlos III, Madrid, Spain; 3 Hospital 12 de Octubre, Instituto de Investigación i+12, Madrid, Spain; University of Dundee, United Kingdom

## Abstract

**Background:**

Clinical audits have emerged as a potential tool to summarize the clinical performance of healthcare over a specified period of time. However, the effectiveness of audit and feedback has shown inconsistent results and the impact of audit and feedback on clinical performance has not been evaluated for COPD exacerbations. In the present study, we analyzed the results of two consecutive nationwide clinical audits performed in Spain to evaluate both the in-hospital clinical care provided and the feedback strategy.

**Methods:**

The present study is an analysis of two clinical audits performed in Spain that evaluated the clinical care provided to COPD patients who were admitted to the hospital for a COPD exacerbation. The first audit was performed from November–December 2008. The feedback strategy consisted of personalized reports for each participant center, the presentation and discussion of the results at regional, national and international meetings and the creation of health-care quality standards for COPD. The second audit was part of a European study during January and February 2011. The impact of the feedback strategy was evaluated in term of clinical care provided and in-hospital survival.

**Results:**

A total of 94 centers participated in the two audits, recruiting 8,143 admissions (audit 1**∶**3,493 and audit 2**∶**4,650). The initially provided clinical care was reasonably acceptable even though there was considerable variability. Several diagnostic and therapeutic procedures improved in the second audit. Although the differences were significant, the degree of improvement was small to moderate. We found no impact on in-hospital mortality.

**Conclusions:**

The present study describes COPD hospital care in Spanish hospitals and evaluates the impact of peer-benchmarked, individually written and group-oral feedback strategy on the clinical outcomes for treating COPD exacerbations. It describes small to moderate improvements in the clinical care provided to COPD patients with no impact on in-hospital mortality.

## Introduction

The existence of a gap between the healthcare that patients actually receive and the guidelines for that healthcare is now well acknowledged [1]. The variations in clinical practice establish a complex interplay of different factors that impact the resulting outcomes in ways that cannot be explained solely by patient characteristics [2]. In this scenario, clinical audits have emerged as a potential tool to summarize the clinical performance of healthcare over a specified period of time. The aim of a clinical audit is to provide health professionals with information that they can use to assess and adjust their performance [3], improving the clinical care provided to patients and the clinical outcomes. Accordingly, the information obtained in an audit should be used to improve care, and several pathways for this aim have been described [4].

In this context, feedback from the audited information constitutes a key step to improve clinical practice [5]. A key question is how successful audit and feedback are in motivating health professionals to modify their clinical practice. To date, several systematic reviews have assessed the effectiveness of audit and feedback with inconsistent results [6], [7]. A recent Cochrane systematic review concluded that audit and feedback generally lead to small, but potentially important, improvements in health care that depend on both the baseline performance and how the feedback is provided [8]. Accordingly, the impact of audit and feedback should be monitored by auditing clinical practices after implementing an intervention [3]; however, it should be acknowledged that the potential effect may also be influenced by the characteristics of the studied disease.

Chronic obstructive pulmonary disease (COPD) is a major and growing health problem [9]. Patients with COPD often suffer episodes of exacerbation during the course of their disease that often require hospitalization; these exacerbations are associated with significant mortality and morbidity [10], [11] and are responsible for most of the social and economic burden of COPD [12]. Thus, the clinical care provided to patients who are admitted to the hospital for a COPD exacerbation should be carefully evaluated. However, the impact of audit and feedback on clinical performance has not been evaluated for COPD exacerbations.

In recent years, Spain had used an auditing process for COPD, named AUDIPOC, which has resulted in the completion of two major clinical audits in the country [13], [14]. In the present study, we evaluated the results of two consecutive clinical audits performed in Spain to assess the clinical care provided to patients who were admitted to the hospital with a physician discharge diagnosis of a COPD exacerbation. The analysis will compare the performances in both audits to evaluate the components of the feedback approach that enhance COPD clinical performance.

## Methods

The present study is an analysis of two clinical audits performed in Spain that evaluated the clinical care provided to COPD patients who were admitted to the hospital for a COPD exacerbation. Both audits had a similar methodology that has been extensively reported in previous publications [13], [14]. Briefly, both studies were clinical audits with prospective case ascertainment of consecutive exacerbation hospital admissions and retrospective data gathering from medical records. All cases admitted to the hospital in any Department or Unit during a 2-month period with a discharge diagnosis of COPD exacerbation were included. The inclusion of a case in the audit was finally decided upon discharge if the diagnosis of COPD exacerbation was included in the discharge report as the cause of admission. The cases with a specific diagnosis that was different from a COPD exacerbation upon admission, including pulmonary edema, pneumonia, pulmonary embolism, pneumothorax, rib fractures, aspiration, pleural effusion or any other associated respiratory or non-respiratory condition, were excluded. The medical records of the included patients were reviewed, and the audit data were extracted. The survivals were followed-up for 90 days from hospitalization to evaluate their vital status and whether the patient had been readmitted. The participant investigators were asked to complete a resource and organization database recording the hospital and respiratory unit or department resources.

The first audit was conducted from November–December 2008, and 129 hospitals participated, recruiting 5,178 patients. The second audit was part of a European study in which 432 centers from 13 countries recruited 18,016 patients. In Spain, 94 hospitals participated and recruited a total of 5,271 cases during January–February 2011. For the purpose of the present study, we selected the centers that participated in both audits. Ninety-four centers were included in this analysis. Altogether, a total of 8,143 cases were included in both audits.

The recorded variables have been reported and included information on the patient characteristics (e.g., age and gender), disease characteristics (e.g., disease severity, clinical features and exacerbation), resources available (e.g., hospital structure, hospital materials and human resources) and clinical practice (e.g., the adopted diagnostic and therapeutic interventions and the adjustment to clinical guidelines). Adherence to the clinical practice guidelines was analyzed following the GOLD recommendations for exacerbation management, which were available at the time of each audit. An adequate use of mechanical ventilation or antibiotics was considered to be present when a patient with such prescriptions correctly received them or when a patient who did not have such prescriptions did not receive them accordingly.

After the first audit, feedback was planned in three different ways. First, a specific report was created for each participant center. In this report, the value of each recorded variable was presented for that center and benchmarked against the regional and national values. Second, the results of the audit were presented in the Spanish Society of Pneumology and Thoracic Surgery (SEPAR) annual congress and in the European Respiratory Society (ERS) annual meeting, and all investigators were invited to attend. Additionally, several smaller local meetings were planned during the next two years following the first audit to communicate the results. Notably, investigators were encouraged to organize meetings at the regional level to discuss the audit results, and several meetings were organized. Third, after the first audit, the SEPAR organized a working committee to create COPD healthcare quality standards, which were made available to all respiratory physicians [15]. All these initiatives were performed during 2009 and 2010, and the second audit was conducted in 2011. After the first audit, participants were not informed of an upcoming second audit.

In Spain, both audits were approved by the institutional review board of Hospital Universitario Virgen del Rocío (approval acta 07/2008 and 16/2010) and confirmed by each participating hospital [13], [16]. Additionally, the hospital management director of each center authorized the audit and agreed not to inform the medical staff that the audit was being conducted so that their medical practice would not change. According to ethical regulations, all data were de-identified in the database by an audit number that was not related to the medical record number or to any personal data. There was no personal information in the database that could be used to identify the patient.

### Statistical analysis

The statistical computations were performed with the Statistical Package for Social Sciences (SPSS, IBM Corporation, Somers, NY) version 18.0. Variables were characterized by the mean and standard deviation or the absolute and relative frequencies of their categories. For describing the centers, we calculated the inter-regional range (IRR), which indicates the region with the highest or lowest mean value for a particular variable. Inferential studies comparing both audits were analyzed with the unpaired Student’s *t*-test (evaluating the variance equivalence with the Levene’s test) or chi-squared test (with the Fisher’s exact test, if required). Kaplan-Meier curves were constructed that evaluated in-hospital survival using the log-rank test to compare the in-hospital global mortality in both audits. The alpha error was set at 0.05.

## Results

Ninety-four Spanish hospitals participated in both clinical audits. The main characteristics of the participant hospitals are summarized in table 1. The participating hospitals were largely public hospitals, with a high proportion being university/teaching hospitals and having a respiratory ward or team. The distribution of the participating hospitals among the different administrative regions is given in table S1 of the online supplement. All the regions in the country participated in this study.

**Table 1:** General characteristics of the hospitals participating in both audits.

Variables	Mean	IRR	p value*
**General characteristics**			
Number of beds (n)	555.3	273–1073	0.049
Catchment population (inhabitants)	324.344	149.799–705.727	NS
University/teaching hospital (%)	62	0–100	NS
Public hospital (%)	98.7	66.7–100	NS
Hospital with intensive care unit (%)	89.9	50–100	0.020
Number of beds in intensive care unit (n)	15	4–26	NS
Spirometry available (%)	100	100–100	NS
Hospital with a respiratory ward (%)	78.5	50–100	NS
Hospital with a respiratory team (%)	96.2	75–100	NS
**Material resources**			
Respiratory outpatient clinic (%)	100	100–100	NS
COPD outpatient clinic (%)	59.5	0–100	NS
Specialty triage system (%)	8.9	0–100	0.039
Emergency department (%)	86.1	66.7–100	NS
Intermediate care unit (%)	30.4	0–100	NS
Number of beds in intermediate care unit (n)	7.8	4–30	NS
Offer non-invasive ventilation for acidosis (%)	96.2	66.7–100	NS
Offer invasive ventilation for acidosis (%)	81.0	50–100	NS
**Human resources**			
Chest physicians (n)	8.7	5–16	0.071
Chest physicians per 1000 beds (n)	17.3	1.2–27.2	0.001
Respiratory trainees (n)	3.6	1.25–7.5	0.025
Respiratory trainees per 100 beds (n)	5.9	2.4–26	<0.001
Physiotherapists (n)	1.4	0.25–3	<0.001
Physiotherapists per 1000 beds (n)	3.1	0.3–15.4	<0.001
Specialist nurses (n)	4.5	0–21.3	<0.001
Specialist nurses per 100 beds (n)	7.1	0–20.4	<0.001
Lung function technicians (n)	2.1	1.4–2.3	0.046
Lung function technicians per 1000 beds (n)	4.8	1.1–11.4	0.011
**Hospital performance**			
Admissions for any cause in the previous year (n)	62.802.5	25.142–154.244	0.044
Percentage of COPD admissions in the unit (%)	57.9	29–83	NS
Daily respiratory physician on call (%)	25.3	0–100	NS
Number of ward rounds (n)	1.5	1–3.1	NS
Percentage of patients seen by physiotherapist (%)	21.9	0–90	NS
Percentage of patients seen by respiratory physician (%)	54.6	30–95	NS
Capacity to perform NIMV on all eligible patients (%)	56.6	0–100	NS
Capacity to perform IMV on all eligible patients (%)	76.6	50–100	NS
Early discharge program (%)	20.3	0–100	<0.001
Percentage of patients in the early discharge program (%)	20.4	5–40	NS
Ability to care for long-term oxygen therapy patients (%)	97.5	66.7–100	NS
Ability to care for home-ventilated patients (%)	89.9	66.7–100	NS
Percentage of patients with rehabilitation (%)	29.7	10–100	0.021

Data are expressed as the mean or relative frequency according to the nature of the variable. IRR: Inter-regional range. NS: not significant.

*p value for the differences between the Spanish regions calculated by the ANOVA or chi-square test.

A total of 8,143 admissions were analyzed from the two audits (audit 1: 3,493; audit 2: 4,650). The characteristics of the patients are summarized in table 2. The cases were mostly males in their eighth decade of life with a high proportion of active smokers. The number of patients with no information on their forced expiratory volume in one second (FEV_1_) was 3,273, including 2,874 patients without spirometry and 399 patients with some spirometric information but no FEV_1_. Altogether, the availability of the FEV_1_ significantly improved in the second audit. The majority of these patients were considered to have severe or very severe disease. More than 40% of the patients had not been previously admitted, and this percentage increased significantly in the second audit. Although there were some statistical differences, the clinical presentation according to the three Anthonisen criteria was not clinically different between the audits.

**Table 2:** Characteristics of the patients included in each audit.

	Audit 1 (n = 3,493)	Audit 2 (n = 4,650)	p value*
Age (years)	73.3 (10.05)	72.7 (10.6)	0.007
Males (n)	3,036 (86.9)	4,001 (86.0)	NS
Tobacco			
• Current smokers (n)	772 (22.1)	1,129 (24.3)	0.023
• Ex-smokers (n)	2,069 (59.2)	3,022 (65.0)	<0.001
• Never smokers (n)	151 (4.3)	211 (4.5)	NS
• Missing (n)	501 (14.3)	288 (6.2)	<0.001
Tobacco history (pack-years)	55.4 (29.6)	51.8 (32.6)	<0.001
Comorbidities (Charlson)	2.8 (1.8)	1.7 (1.7)	<0.001
Body mass index (kg/m^2^)	27.6 (5.3)	27.7 (5.4)	NS
Spirometry: FEV_1_ (%)	44.8 (17.0)	45.1 (16.4)	NS
GOLD spirometric assessment:			
• No information available	1,812 (51.9)	1,461 (31.4)	<0.001
• No obstruction	157 (4.5)	247 (5.3)	NS
• Mild (n)	50 (1.4)	74 (1.6)	NS
• Moderate (n)	422 (12.1)	887 (19.1)	<0.001
• Severe (n)	737 (21.1)	1,424 (30.6)	<0.001
• Very severe (n)	315 (9.0)	557 (12.0)	<0.001
First admission (n)	1,415 (40.5)	2,093 (45.0)	<0.001
Respiratory ward (n)	1,921 (55.5)	2,637 (56.7)	NS
Dyspnea increase (n)	3,328 (95.3)	4,431 (95.3)	NS
Sputum increase (n)	2,258 (64.6)	3,140 (67.5)	0.004
Sputum color change (n)	1,654 (47.4)	2,349 (50.5)	0.002

Data are expressed as the mean (standard deviation) or absolute (relative) frequencies. NS: not significant. FEV_1_: forced expiratory volume in one second.

*Calculated using the unpaired Student’s *t*-test or chi-square test.

The diagnostic procedures improved by the second audit (table 3). The proportion of cases with blood gas analysis significantly increased from 88.5 to 90.9%, and radiographies were also slightly more frequently performed in the second audit than in the first (99.5% vs. 97.2%). Although the increases were significant, they were not extremely different. The severity of the blood gas alterations was similar between the audits. An unexpected finding was the high proportion of consolidations in the radiographies for the second audit.

**Table 3:** Diagnostic procedures performed during admission in each audit.

	Audit 1 (n = 3,493)	Audit 2 (n = 4,650)	p value*
Blood gas analysis performed (n)	3,091 (88.5)	4,227 (90.9)	<0.001
With oxygen (n)	621 (17.8)	1,181 (26.1)	<0.001
pH	7.39 (0.06)	7.39 (0.07)	NS
PaO_2_ (mmHg)	58.9 (15.0)	60.1 (21.5)	0.007
PaCO_2_ (mmHg)	48.5 (14.7)	48.5 (15.8)	NS
Bicarbonate (mEq/l)	28.7 (5.1)	28.6 (5.2)	NS
Acidosis (n)	549 (15.7)	737 (15.8)	0.002
Radiography performed (n)	3,396 (97.2)	4,627 (99.5)	<0.001
Consolidation (n)	12 (0.3)	384 (8.3)	<0.001
Interstitial (n)	20 (0.6)	152 (3.3)	<0.001
Neoplasm (n)	53 (1.5)	66 (1.4)	NS
Pleural effusion (n)	119 (3.4)	161 (3.5)	NS
Pneumothorax (n)	2 (0.1)	6 (0.1)	NS

Data are expressed as the mean (standard deviation) or absolute (relative) frequencies. NS: not significant. PaO_2_: partial pressure of oxygen in arterial blood. PaCO_2_: partial pressure of carbon dioxide in arterial blood.

*Calculated using the unpaired Student’s *t*-test or chi-square test.

The therapeutic interventions before admission, during hospitalization and at discharge are summarized in table 4. Before admission, the number of administered treatments increased in the second audit, with a special emphasis on short-acting bronchodilators, long-acting muscarinic antagonists (LAMA) and systemic steroids. During admission, there were also some differences between the audits, but they were less striking. Notably, there was a significant decrease in systemic steroid use and a slight decrease in oxygen use in the second audit. The therapeutic recommendations at discharge were similar. Although the differences were significant, the percentages of each therapeutic option were not very dramatic.

**Table 4:** Therapeutic interventions performed in both audits.

	Previous admission			During admission			At discharge		
	Audit 1 (n = 3,493)	Audit 2 (n = 4,650)	p value*	Audit 1 (n = 3,493)	Audit 2 (n = 4,650)	p value*	Audit 1 (n = 3,493)	Audit 2 (n = 4,650)	p value*
SABA (n)	1,227 (35.1)	2,183 (46.9)	<0.001	3,069 (87.9)	4,184 (90.0)	0.003	1,703 (48.8)	1,894 (40.7)	<0.001
SAMA (n)	587 (16.8)	1,022 (22.0)	<0.001	3,062 (87.7)	4,333 (93.2)	<0.001	780 (22.3)	834 (17.9)	<0.001
LABA alone (n)	186 (5.3)	265 (5.7)	<0.001	–	–	–	229 (6.6)	339 (7.3)	<0.001
LAMA (n)	1,286 (36.8)	2,446 (52.6)	<0.001	–	–	–	2,243 (64.2)	3,055 (65.7)	<0.001
ICS alone (n)	277 (7.9)	301 (6.5)	<0.001	448 (12.8)	844 (18.2)	<0.001	284 (8.1)	278 (6.0)	<0.001
ICS/LABA combination (n)	1,529 (43.8)	2,819 (60.6)	<0.001	–	–	–	2,584 (74.0)	3,487 (75.0)	<0.001
Methylxanthines (n)	235 (6.7)	412 (8.9)	<0.001	347 (9.9)	113 (2.4)	<0.001	336 (9.6)	417 (9.0)	<0.001
Systemic steroids (n)	415 (11.9)	771 (16.6)	<0.001	3,185 (91.2)	4,123 (88.7)	<0.001	2,407 (68.9)	2,908 (62.5)	<0.001
Antibiotics (n)	524 (15.0)	847 (18.2)	<0.001	3,129 (89.6)	4,309 (92.7)	<0.001	1,808 (51.8)	2,284 (49.1)	<0.001
Oxygen (n)	–	–	–	3,355 (97.4)	4,422 (96.5)	0.009	1,474 (42.2)	1,892 (40.7)	<0.001
Non-invasive ventilation (n)	–	–	–	353 (10.1)	555 (11.9)	<0.001	210 (6.0)	318 (6.8)	<0.001
Invasive ventilation (n)	–	–	–	28 (0.8)	80 (1.7)	<0.001	–	–	–

Data are expressed as the mean (standard deviation) or absolute (relative) frequencies. NS: not significant. SABA: Short-acting beta agonist, SAMA: short-acting muscarinic antagonist. LABA: long-acting beta agonist, LAMA: long-acting muscarinic antagonist, ICS: inhaled corticosteroid.

*Calculated using the unpaired Student’s *t*-test or chi-square test. The figures for non-invasive ventilation at discharge refer to home mechanical ventilation. –: not applicable or not recorded.

The 10 recommendations evaluated according to the GOLD guidelines are summarized in table 5. The majority of these recommendations improved in the second audit. However, although the differences were significant, they were not very striking.

**Table 5:** Adjustment to the GOLD guidelines.

	Audit 1 (n = 3,493)	Audit 2 (n = 4,650)	p value*
Spirometry performed	2,066 (59.1)	3,202 (68.9)	<0.001
Blood gas analysis performed	3,091 (88.5)	4,227 (90.9)	<0.001
Radiography performed	3,396 (97.2)	4,627 (99.5)	<0.001
Treatment with oxygen	3,355 (97.4)	4,422 (96.5)	0.009
Short-acting bronchodilator use	3,337 (95.5)	4,473 (96.2)	NS
No methylxanthine use	3,146 (90.1)	4,537 (97.6)	<0.001
Systemic steroids	3,185 (91.2)	4,123 (88.7)	<0.001
Adequate antibiotic use	1,930 (61.5)	2,782 (60.3)	NS
Adequate NIMV use	2,625 (85.1)	3,491 (85.6)	NS
Adequate IMV use	2,994 (97.1)	3,906 (95.8)	0.005

Data are expressed as the mean (standard deviation) or absolute (relative) frequencies. NS: not significant. NIMV: non-invasive mechanical ventilation. IMV: invasive mechanical ventilation.

*Calculated using the unpaired Student’s *t*-test or chi-square test.

**Figure 1:**
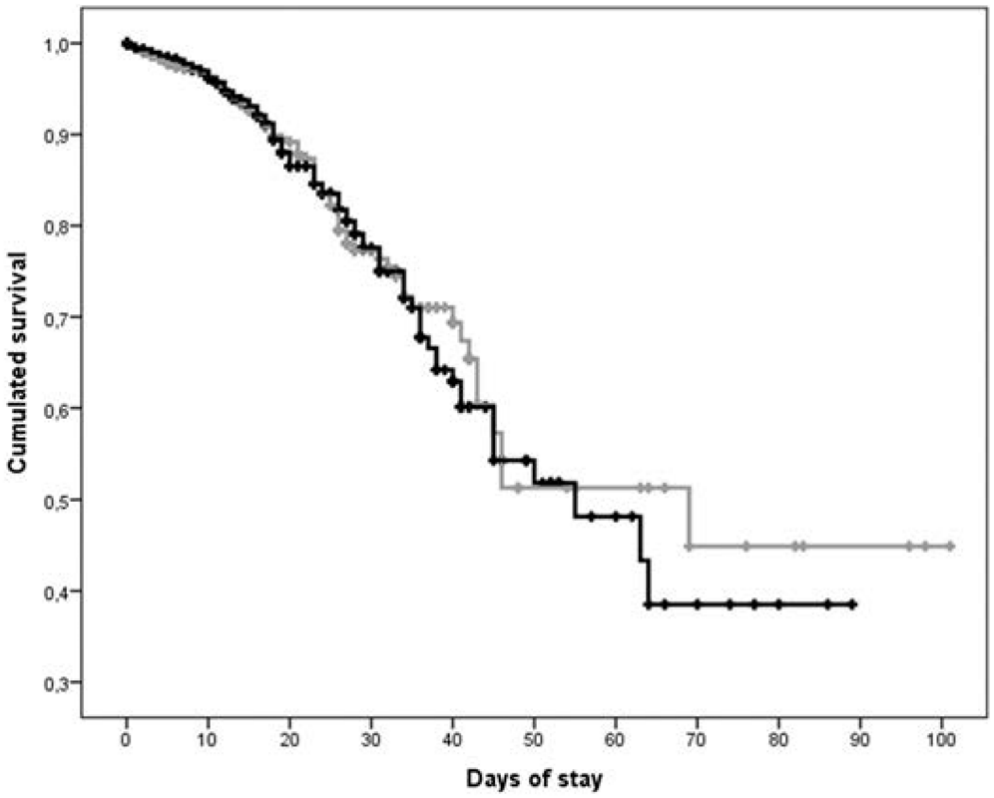
Kaplan-Meier curves comparing the in-hospital survival between the audits. The grey line represents the first audit. The black line represents the second audit.

The survival curves for both audits are shown in figure 1. The in-hospital mortality was not different between the two audits. The first audit reported 164 (4.7%) deaths, and the second reported 202 (4.3%) deaths, and this difference was not significantly different. Accordingly, the median survival in the Kaplan-Meier analysis was not significantly prolonged in the second audit (56.7 days) compared with the first audit (64.6 days). The lengths of stay were very similar between the two audits, with 8.7 (7.8) days in audit 1 and 8.5 (7.3) days in audit 2 (not significantly different).

## Discussion

The present study describes the state of COPD hospital care in Spanish hospitals and evaluates the impact of a feedback strategy on clinical care. After two consecutive audits, with feedback performed in between audits, we have observed some improvements in the clinical care provided to COPD patients. However, the majority of these improvements were small or moderate and did not impact the in-hospital mortality. This finding suggests that performing a clinical audit and providing the participant centers with the results according to our feedback strategy increased awareness and improved some aspects of COPD exacerbations, although there was no clear benefit on the outcomes.

Clinical audits have gained traction in healthcare systems as a way of obtaining information on the clinical care being provided. This information is of interest both for people funding healthcare, who want to ensure that the care they purchase is of the highest possible standard, and for patients who hope to receive safe and effective healthcare. However, despite evidence from many studies on audit and feedback, there is still limited information on how to use these data. A recent systematic review assessed the effectiveness of audit and feedback and reported an inconsistent picture; some evaluations obtained positive results, whereas others did not [8]. Interestingly, although previous studies suggest that audit and feedback may improve the performance of health care providers, the effects are generally moderate or small [17]. In our study, we also found moderate or small improvements in the clinical performance for COPD. Notably, audit and feedback measures are most likely to be beneficial when the existing practice is farthest away from what is desired and when the feedback is more intensive. However, the initial situation in our study was not far from the current guidelines; therefore, the feedback effect may be limited by a ceiling effect. The rationale for this limitation is most likely because clinical care for COPD exacerbations does not require complex interventions. Additionally, the recommendations for clinical care are closely followed in the medical community in our country, and the current guidelines are well known [18]. However, in this case, even small to moderate improvements in quality are worthwhile. COPD is a dramatic disease with a high prevalence [19], high in-hospital mortality and high readmission rate [16], and adequate clinical care can influence its outcome. Therefore, any effort to improve clinical care for these patients is worthwhile. Accordingly, some examples should be highlighted. The use of spirometry for diagnosis was improved in the second audit (table 2). Spirometry is a simple, non-invasive diagnostic procedure that is a necessary pillar for diagnosing COPD. In Spain, the use of spirometry has recently been evaluated, showing significant bottlenecks in primary and secondary care [20]. Thus, improvements in the use of spirometry will contribute to a better diagnosis and identification of these patients. In this regard, it is worth noting that 4.5% of patients in the first audit and 5.3% of those in the second audit did not show an obstructive pattern on spirometry. Therefore, such patients should not have been diagnosed with COPD and consequently COPD exacerbations. According to current guidelines, the diagnosis of COPD exacerbations should be solely based on clinical symptoms. However, such definition may be problematic and has been recently challenged [21]. Notably, in this study we were auditing the clinical care provided to patients deemed to have COPD exacerbations by the clinician in charge and thus treated accordingly. Arterial blood gas analysis was increased by 2.4%. The performance of a blood gas analysis is one of the key diagnostic measures for evaluating the severity of an exacerbation in hospitals. In those centers that do not offer this procedure, the alternative is to use pulse-oximetry to evaluate oxygen saturation. However, this method does not measure the pH or PaCO_2_, potentially leading to an incorrect assessment [22]. The use of chest radiography is another diagnostic intervention that is needed to eliminate other potential accompanying conditions that may mimic or worsen the exacerbation. In this regard, there is an ongoing debate on the significance of the consolidation frequently found in these COPD patients [23]. Additionally, from a therapeutic standpoint, several positive changes were observed after the feedback strategy was implemented. Short-acting bronchodilators were more frequently used, and the use of methylxanthines, which are no longer recommended, was decreased. Interestingly, the use of systemic steroids was also decreased, which is an unexplained finding that will need to be addressed in the future. The adequate use of invasive mechanical ventilation will also need to be further explored. However, all of these measures had a negligible impact on in-hospital mortality or length of stay and several factors are deemed to play a role in determining such clinically relevant endpoints. One potential explanation for such a finding is the presence of a ceiling effect with a good starting position for the majority of the items. Previous studies have demonstrated the influence of the baseline performance on the potential improvement gained [24]. Another explanation may be related to the feedback strategy used. Although it seems intuitive that health care professionals will modify their clinical practice if they receive feedback that their clinical practice is inconsistent with those of their peers or the accepted guidelines, this outcome has not been consistently demonstrated. Although there is some controversy that feedback with peer comparison is either more or less effective than other initiatives [25], [26], we provided feedback that was benchmarked against the peer average because providing this information may help participants understand the relative deviation of their own measures, which could improve patient outcomes.

Feedback can be delivered in different ways, and audit and feedback can be used as components of a multifaceted strategy to improve the quality of healthcare. In our study, we provided oral and written information at the individual level and oral information at the group level. Additionally, healthcare quality standards for COPD were constructed according to the experience with the first AUDIPOC audit [15]. Although this feedback strategy informs the participants of the audit results, alternative feedback strategies may result in different outcomes. For example, there are care bundles, including several evidence-based practices, that should be delivered to all patients to guarantee a set of minimum requirements for clinical care and several initiatives for COPD have been implemented [27], [28].

Some limitations must be considered when interpreting our results. Within each region, the participating hospitals volunteered. Therefore, although all regions were sampled, there was no attempt at representative sampling. As a consequence, the hospitals involved in the study were different in organizational terms and there was a certain degree of variability concerning the available resources (Table 1). However, the participating centers were identical for both audits. Additionally, the investigators were the same for the majority of the involved centers. We therefore believe that the potential impact of structural differences between the first and the second audits should be minimal. Another limitation is the considerably high number of investigators who recorded the information using different information sources, as is intrinsically associated with audits.

In summary, our study evaluated the impact of a peer-benchmarked, individually written and group-oral feedback strategy on the clinical outcomes for treating COPD exacerbations. The results of our study suggest that performing a clinical audit and providing the participant centers with the results according to our feedback strategy increased awareness and improved some aspects of COPD exacerbation treatment. Accordingly, other feedback strategies may yield different results. Although we did not observe a clear benefit in the clinical outcomes, several aspects of the diagnostic and therapeutic clinical care provided to the COPD patients admitted to hospitals seemed to improve, which, in turn, may reduce the gap between the healthcare that patients receive and the guidelines for that care.

## Supporting Information

Table S1 **Percentual distribution of the cases included in both audits according to the region within the country.**
(DOCX)

File S1 **Membership of the AUDIPOC and the European COPD Audit studies.**
(DOCX)
