# Supplementary material for: Results from an Audit Feedback Strategy for Chronic Obstructive Pulmonary Disease In-Hospital Care: A Joint Analysis from the AUDIPOC and European COPD Audit Studies
Source: PLoS One. 2014 Oct 15;9(10):e110394. doi: 10.1371/journal.pone.0110394 (PMC4198296; doi:10.1371/journal.pone.0110394)
Supplement: Table S1 — Percentual distribution of the cases included in both audits according to the region within the country. (DOCX) [file pone.0110394.s001.docx]

Table S1. Percentual distribution of the cases included in both audits according to the region within the country.

| Region | Number of centers  (n=94) | Number of patients | | |
| --- | --- | --- | --- | --- |
|  |  | Total  (n=8,143) | Audit 1  (n=3,493) | Audit 2  (n=4,650) |
| Andalucía | 12 (12.8) | 811 (10.0) | 320 (9.2) | 491 (10.6) |
| Aragón | 3 (3.2) | 284 (3.5) | 137 (3.9) | 147 (3.2) |
| Asturias | 3 (3.2) | 551 (6.8) | 208 (6.0) | 343 (7.4) |
| Baleares | 3 (3.2) | 380 (4.7) | 151 (4.3) | 229 (4.9) |
| Canarias | 1 (1.1) | 60 (0.7) | 15 (0.4) | 45 (1.0) |
| Cantabria | 3 (3.2) | 482 (5.3) | 224 (6.4) | 204 (4.4) |
| Castilla La Mancha | 4 (4.3) | 487 (6.0) | 232 (6.6) | 255 (5.5) |
| Castilla León | 8 (8.5) | 575 (7.1) | 251 (7.2) | 324 (7.0) |
| Cataluña | 9 (9.6) | 532 (6.5) | 213 (6.1) | 319 (6.9) |
| Extremadura | 3 (3.2) | 205 (2.5) | 77 (2.2) | 128 (2.8) |
| Galicia | 3 (3.2) | 398 (4.9) | 132 (3.8) | 266 (5.7) |
| La Rioja | 1 (1.1) | 212 (2.6) | 115 (3.3) | 97 (2.1) |
| Madrid | 18 (19.1) | 1,097 (13.5) | 465 (13.3) | 632 (13.6) |
| Murcia | 3 (3.2) | 158 (1.9) | 76 (2.2) | 82 (1.8) |
| Navarra | 1 (1.1) | 118 (1.4) | 27 (0.8) | 91 (2.0) |
| País Vasco | 10 (10.6) | 1,047 (12.9) | 468 (13.4) | 579 (12.5) |
| Valencia | 9 (9.6) | 800 (9.8) | 382 (10.9) | 418 (9.0) |

Values expressed as the absolute and relative frequencies according to the total number of cases or centers.
