# Supplementary material for: Results from an Audit Feedback Strategy for Chronic Obstructive Pulmonary Disease In-Hospital Care: A Joint Analysis from the AUDIPOC and European COPD Audit Studies
Source: PLoS One. 2014 Oct 15;9(10):e110394. doi: 10.1371/journal.pone.0110394 (PMC4198296; doi:10.1371/journal.pone.0110394)
Supplement: File S1 — Membership of the AUDIPOC and the European COPD Audit studies. (DOCX) [file pone.0110394.s002.docx]

# Steering committees

| Audit 1: AUDIPOC | Audit 2: ERS COPD audit |
| --- | --- |
| **STEERING COMMITTEE:**  POZO RODRIGUEZ, Francisco.  CASTRO ACOSTA, Ady Angélica.  BOUKICHOU ABDELKADER, Nisa  ESTEBAN GARCIA-NAVAS, Sara  **SCIENTIFIC COMMITTEE:**  POZO RODRÍGUEZ, Francisco  AGUSTÍ, Alvar  ÁLVAREZ MARTÍNEZ, Carlos José  CAPELASTEGUI SAIZ, Alberto  ESTEBAN GONZÁLEZ, Cristóbal  HERNÁNDEZ CARCERENY, Carmen  IZQUIERDO ALONSO, José Luis  LOPEZ CAMPOS, José Luis  MELERO MORENO, Carlos | **EUROPEAN STEERING COMMITTEE:**  ROBERTS, C. Michael  HARTL, Sylvia  LOPEZ-CAMPOS, Jose Luis  **SPANISH STEERING COMMITTEE:**  POZO RODRÍGUEZ, Francisco  ANCOCHEA, Julio  CASTRO ACOSTA, Ady Angélica  ÁLVAREZ MARTÍNEZ, Carlos José  LOPEZ-CAMPOS, Jose Luis  **DATA ANALYSIS TEAM**  POZO RODRÍGUEZ, Francisco  LOPEZ-CAMPOS, Jose Luis  CASTRO ACOSTA, Ady Angélica  ABRAIRA-SANTOS, Victor  LOPEZ-QUILEZ, Antonio  DORADO, Juan |

# Regional coordinators

|  | Audit 1: AUDIPOC | Audit 2: ERS COPD audit |
| --- | --- | --- |
| Andalucía | José Luis López-Campos | José Luis López-Campos |
| Aragón | José Manuel Gascón Pelegrín  Anselmo López  Mónica Torrijos | Luis Borderías Clau |
| Asturias | Cristina Martinez | Cristina Martinez |
| Baleares | Borja García-Cosío | Borja García-Cosío |
| Canarias | José Gabriel Julia | Ana Velázquez Benítez |
| Cantabria | Ramón Agüero Balbín | Ramón Agüero Balbín |
| Castilla La Mancha | Jesús Fernández Francés | Jose Celdran Gil |
| Castilla León | Jesús Reyes Hernández Hernández | Jesús Reyes Hernández Hernández |
| Cataluña | Carlos Martínez Rivera  Eduardo Monsó Molas | Carlos Martínez Rivera |
| Extremadura | Juan Antonio Riesco Miranda | Juan Antonio Riesco Miranda  José Antonio Gutiérrez Lara |
| Galicia | Juan Suárez Antelo | Alberto Fernández Villar |
| La Rioja | Manuel Barrón Medrano | Manuel Barrón Medrano |
| Madrid | Carlos Melero Moreno  Carlos José Álvarez Martínez | Julio Ancochea Bermúdez  Carlos Melero Moreno |
| Murcia | Juan Miguel Sánchez Nieto | Juan Miguel Sánchez Nieto |
| Navarra | Javier Hueto Perez de Heredia | Javier Hueto Perez de Heredia |
| País Vasco | Cristóbal Esteban González | Cristóbal Esteban González |
| Valencia | Juan José Soler Cataluña | Pablo Catalán Serra |

# Local investigators and data managers

| Region | Province | Hospital | Audit 1: AUDIPOC | Audit 2: ERS COPD audit |
| --- | --- | --- | --- | --- |
| Andalucía | Almería | Complejo Hospitalario Torrecárdenas | José Calvo Bonachera | José Calvo Bonachera  Maria Paz Martínez Cortes |
| Andalucía | Cádiz | Hospital de la Línea de la Concepción | Armando Falces Sierra | Armando Falces Sierra |
| Andalucía | Cádiz | Hospital General de Jerez de la Frontera | Gregorio Soto Campos | Gregorio Soto Campos  Aida García Cuesta |
| Andalucía | Cádiz | Hospital Universitario Puerta del Mar | Fernando Romero Valero  Isidro Blanco Sáez | Fernando Romero Valero  Isidro Blanco Sáez |
| Andalucía | Córdoba | Hospital Universitario Reina Sofía | Marisol Arenas de la Riva  María Jesús Cobos Ceballos | Marisol Arenas de la Riva |
| Andalucía | Granada | Hospital Universitario San Cecilio | Alicia Conde Valero | Alicia Conde Valero |
| Andalucía | Huelva | Hospital Infanta Elena | Rosa Vázquez Oliva  Fernando Hernández Utrera | Rosa Vázquez Oliva  Fernando Hernández Utrera |
| Andalucía | Jaén | Hospital de Alta Resolución Alto Guadalquivir | Juan Manuel Bravo Santervás | Francisco Canales Cid |
| Andalucía | Jaén | Complejo Hospitalario de Jaén | Bernardino Alcázar Navarrete | Bernardino Alcázar Navarrete |
| Andalucía | Málaga | Hospital Comarcal de Vélez Málaga | Carlos Rueda | Carlos Rueda |
| Andalucía | Sevilla | Hospital Universitario Valme | Inmaculada Alfageme Michavila | Inmaculada Alfageme Michavila  Zulema Palacios Hidalgo |
| Andalucía | Sevilla | Hospital Universitario Virgen del Rocío | Jose Luis López-Campos  Pablo Pérez Navarro | Jose Luis López-Campos  Pablo Pérez Navarro |
| Aragón | Huesca | Hospital San Jorge de Huesca | Luis Borderías Clau | Luis Borderías Clau  Helena Briz Muñoz |
| Aragón | Zaragoza | Hospital Clínico Universitario Lozano Blesa | Joaquín Carlos Costán Galicia | Joaquín Carlos Costán Galicia  Laura Anoro Abenoza |
| Aragón | Zaragoza | Hospital Miguel Servet | Salvador Bello Dronda  Andrés Sánchez Barón  José Manuel Gascón Pelegrín | Salvador Bello Dronda  Andrés Sánchez Barón |
| Asturias | Asturias | Hospital San Agustín de Avilés | Fernando Álvarez Navascues  Marta García Clemente | Fernando Álvarez Navascues  Manuel Villanueva Montes |
| Asturias | Asturias | Hospital de Cabueñes | Teresa Pascual Pascual  Concepción Díaz Sánchez | Teresa Pascual Pascual |
| Asturias | Asturias | Hospital Central de Asturias | Cristina Martínez  Marta García Clemente  Aida Quero Martínez | Cristina Martínez  Marta García Clemente  Aida Quero Martínez  Ana Pando Sandoval  Francisco Julián López González |
| Baleares | Mallorca | Hospital de Manacor | Rosa Maria Irigaray Canals | Rosa Maria Irigaray Canals  Maria José Cons |
| Baleares | Mallorca | Hospital Son Llatzer | Salvador Pons Vivas | Salvador Pons Vivas |
| Baleares | Mallorca | Hospital Son Espases | Borja García-Cosío Piqueras | Borja García-Cosío Piqueras  Rocío Córdova Díaz |
| Canarias | Tenerife | Hospital Nuestra Señora de la Candelaria | Magdalena Alonso  Ruth Pitti  José Batista  Orlando Acosta Fernández | Magdalena Alonso  Ruth Pitti  Luisa Eiroa González  Ana Velázquez |
| Cantabria | Cantabria | Hospital Comarcal de Laredo | Miguel Zabaleta Murguiondo | Miguel Zabaleta Murguiondo |
| Cantabria | Cantabria | Hospital de Sierrallana | Mar García Pérez | Mar García Pérez |
| Cantabria | Cantabria | Hospital Universitario Marqués de Valdecilla | Ramón Agüero Balbín  Beatriz Abascal Bolado | Ramón Agüero Balbín  Beatriz Abascal Bolado  Carlos Amado Diago |
| Castilla La Mancha | Cuenca | Hospital Virgen de la Luz | Mª José Peirón Puyal | Mª José Peirón Puyal  José Maria Peñas Herrero  Maria Eugenia Casado López  Rosario Vargas Gonzalez |
| Castilla La Mancha | Guadalajara | Hospital Universitario de Guadalajara | Jesús Fernández Francés  José Luis Izquierdo | Jesus Fernández Francés  Elisabeth Guzmán Robles  Juan Pablo Rodríguez Gallego |
| Castilla La Mancha | Toledo | Hospital Nuestra Señora del Prado | José Celdrán Gil | José Celdrán Gil |
| Castilla La Mancha | Toledo | Hospital Virgen de la Salud | Encarnación López Gabaldón | Encarnación López Gabaldón |
| Castilla León | Ávila | Hospital Nuestra Señora de Sonsoles | Eugenio Trujillo Santos  José Eugenio Alonso Muñoz | Jesús Reyes Hernández Hernández  José Eugenio Alonso Muñoz |
| Castilla León | Burgos | Hospital Santos Reyes | Pedro Cancelo Suárez | Pedro Cancelo Suárez |
| Castilla León | León | Hospital del Bierzo | Juan Ortiz De Saracho y Bobo | Juan Ortiz De Saracho y Bobo |
| Castilla León | Palencia | Complejo Asistencial de Palencia | María Ángeles Fernández Jorge | Maria Ángeles Fernández Jorge |
| Castilla León | Segovia | Hospital General de Segovia | Graciliano Estrada Trigueras | Graciliano Estrada Trigueras |
| Castilla León | Soria | Complejo Asistencial de Soria, Hospital Santa Bárbara | José Luis Orcastegui Candial | José Luis Orcastegui Candial  Isabel Ramos Cancelo  Ruth García García |
| Castilla León | Valladolid | Hospital Universitario de Valladolid | Carlos Disdier  Enrique Macías Fernández | Carlos Disdier  Enrique Macías Fernández  Jaime Sanabria  Angela Peñaloza |
| Castilla León | Valladolid | Hospital Universitario Rio Hortega | Félix del Campo Matías | Félix Del Campo Matías |
| Cataluña | Barcelona | Hospital Comarcal del Alto Penedés | Nuria Rodríguez Lázaro | Nuria Rodríguez Lázaro |
| Cataluña | Barcelona | Parque Sanitario San Juan de Dios | Luis Lores Obradors | Luis Lores Obradors |
| Cataluña | Barcelona | Hospital del Mar | Joaquín Gea Giral  Rosa Pedreny  Sergio Pascual | Joaquín Gea Giral  Rosa Pedreny  Sergio Pascual |
| Cataluña | Barcelona | Hospital Universitario Germans Trias i Pujol | Ignacio Garcia Olivé  Eduardo Monso Solas | Ignacio Garcia Olivé  Carlos Martínez  Ramona Hervas |
| Cataluña | Barcelona | Hospital Clínico Barcelona | Néstor Soler Porcar  Carmen Hernández | Néstor Soler Porcar  Silvia Valls |
| Cataluña | Barcelona | Hospital Universitario Valle de Hebrón | Esther Rodríguez González | Esther Rodríguez González  Eva Tapia Melechon  Ángeles Barrio Guirado  Milagros Gándara Sanz  David Lobillo Lopez  Eugenia Bueno Portela |
| Cataluña | Barcelona | Corporación Sanitaria Parc Taulí | Eduardo Monso Solas | Eduardo Monso Solas  Laia Seto Gort |
| Cataluña | Gerona | Hospital Universitario Doctor Jose Trueta | Manuel Haro Estarriol | Manuel Haro Estarriol |
| Cataluña | Tarragona | Hospital Juan XXIII | Leonardo Esteban | Leonardo Esteban |
| Extremadura | Badajoz | Hospital de Mérida | Germán García de Vinuesa | Germán García de Vinuesa |
| Extremadura | Badajoz | Hospital Universitario Infanta Cristina | José Antonio Gutierrez Lara  Fernando Fuentes Otero | José Antonio Gutiérrez Lara  José Antonio Marín Torrado  Estefania Molina Ortiz  Lourdes Cañón Barroso |
| Extremadura | Cáceres | Hospital San Pedro de Alcántara | Juan Antonio Riesco Miranda | Juan Antonio Riesco Miranda  Elena Badarán  Maria José López Jiménez  Alfonso García Guisado  Mirian Torres Gonzalez |
| Galicia | Orense | Complejo Hospitalario de Orense | Pedro Marcos Velázquez  Carlos Vilariño Pombo  Jose Manuel García Pazos | Pedro Marcos Velázquez  Isaura Parente Lamelas  Mariluz Santalla Martínez |
| Galicia | Pontevedra | Hospital Povisa | Mª Dolores Corbacho Abelaira | Mª Dolores Corbacho Abelaira |
| Galicia | Pontevedra | Complejo Hospitalario Universitario de Vigo | Alberto Fernández Villar  Cristina Represas Represas | Alberto Fernández Villar  Cristina Represas Represas  Maria Isabel Botana |
| La Rioja | La Rioja | Hospital General de la Rioja | Manuel Barrón Medrano  Carlos Ruiz Martínez y | Manuel Barrón Medrano  Carlos Ruíz Martínez  Maria del Carmen Mascareño  Francisco Campano |
| Madrid | Madrid | Hospital Sureste Arganda del Rey | Sergio Salgado Aranda | Sergio Salgado Aranda  Mónica Gómez García  María Piñeiro Martínez |
| Madrid | Madrid | Hospital del Henares | No participo | María Ángeles Ruiz-Cobos  Belén Arnalich Jimenez  Álvaro Casanova Espinosa  Eva de Santiago Delgado |
| Madrid | Madrid | Hospital Infanta Cristina | Beatriz Jara Chinarro  María Teresa Río Ramírez | Beatriz Jara Chinarro |
| Madrid | Madrid | Hospital Infanta Leonor | Maria Jesus Buendía  Carmen Matesanz Ruiz | Maria Jesús Buendía  Carmen Matesanz Ruiz  África Alcorta Mesas  Vanesa Lores Gutiérrez  María Belén López-Muñiz Ballesteros  Julio Hernández Vázquez  Yunelsey Anta Mejías |
| Madrid | Madrid | Hospital Infanta Sofía | Raúl Moreno Zabaleta  Maria Teresa Ramírez Prieto  Blas Rojo Moreno Arrones | Raúl Moreno Zabaleta  María Teresa Ramírez Prieto |
| Madrid | Madrid | Hospital de la Princesa | Enrique Zamora García | Gonzalo Segrelles Calvo  Rosa Mar Gómez Púnter |
| Madrid | Madrid | Fundación Jiménez Díaz | German Peces Barba | German Peces Barba  Sandra Pelícano  José Fernández |
| Madrid | Madrid | Hospital Central de la Defensa | Javier Jareño  Sergio Campos Téllez  Ignacio Granda Uribe | Javier Jareño  Sergio Campos Téllez |
| Madrid | Madrid | Hospital Príncipe de Asturias | Soledad Alonso Viteri | Soledad Alonso Viteri  Alicia Ferreira  Antonio Ruiz  Concepción Losada  Esther Alonso Peces  Gerardo Vázquez  Julio Flores |
| Madrid | Madrid | Hospital de Móstoles | Dolores Álvaro | Dolores Álvaro  Natividad Quílez Ruíz-Rico  Raquel Pérez Rojo  María Vázquez Mezquita  Olga Navarrete  Silvia Sánchez |
| Madrid | Madrid | Hospital Severo Ochoa | Asunción Perpina | Asunción Perpina  Pilar Alba |
| Madrid | Madrid | Hospital Universitario 12 de Octubre | Virginia Pérez González | Virginia Pérez González  Carlos Álvarez Martínez |
| Madrid | Madrid | Hospital Universitario Clínico San Carlos | Gema Rodríguez Trigo | Gema Rodríguez Trigo |
| Madrid | Madrid | Hospital Puerta de Hierro | Antolín López Viña  Rosa Malo de Molina Ruiz  Piedad Ussetti | Antolín López Viña  Rosa Malo de Molina Ruiz  Miriam Aguilar  Patricia Mínguez Clemente  Andrea Trisán Alonso  Manuel Valle Falcones |
| Madrid | Madrid | Hospital de Getafe | Mª Antonia Juretschke Moragues | Mª Antonia Juretschke Moragues Pilar Andres  David Lin |
| Madrid | Madrid | Hospital La Paz | Francisco García Río  Sergio Alcolea Batres | Francisco García Río |
| Madrid | Madrid | Hospital Gregorio Marañón | José Miguel Rodríguez González-Moro  Jorge Eisner Garcia | José Miguel Rodríguez González-Moro  Jorge Eisner Garcia |
| Madrid | Madrid | Hospital Ramón y Cajal | Salvador Diaz Lobato  Esteban Pérez Rodríguez | Salvador Diaz Lobato  Esteban Pérez Rodríguez |
| Murcia | Murcia | Hospital de los Arcos | Damián Malia Alvarado  Nuria Castejón Pina  Jose A Ros Lucas  Ada Luz Andreu Rodríguez | Damián Malia Alvarado  Nuria Castejón Pina  Jose Antonio Ros Lucas  Ada Luz Andreu Rodríguez |
| Murcia | Murcia | Hospital General Universitario Morales Meseguer | Juan Miguel Sánchez Nieto  Roberto Bernabeu Mora  Maria Loreto Alemany Frances | Juan Miguel Sánchez Nieto  Roberto Bernabeu Mora  Manuel Castilla Martínez  Olga Meca Birlanga |
| Murcia | Murcia | Hospital Universitario Santa Lucía | Pilar Berlinches Acin  Inés Bernal Belijar | Pilar Berlinches Acin  Inés Bernal Belijar |
| Navarra | Navarra | Complejo Hospitalario de Navarra | Victor Manuel Eguía Astibia  Jalil Abú-Shams | Víctor Manuel Eguía Astibia  Javier Hueto Pérez de Heredia  Joan Boldu Mitgans  Pilar Cebollero Rivas  José Antonio Cascante Rodrigo  Idoya Pascal Martínez |
| País Vasco | Álava | Hospital Universitario de Álava, Hospital de Txagorritxu | Laura Tomás | Laura Tomás |
| País Vasco | Guipúzcoa | Hospital de Zumárraga | Silvia Dorronsoro  Cristina Estirado  Raquel Sánchez | Silvia Dorronsoro |
| País Vasco | Guipúzcoa | Hospital de Mendaro | Susana Chic Palacin  José Ignacio Royo  Nicolás Gurrutxaga | Susana Chic Palacin  José Ignacio Royo |
| País Vasco | Guipúzcoa | Hospital Alto Deba, Mondragón | Mikel Temprano Gogenola  Iñaki Peña | Mikel Temprano Gogenola  Iñaki Peña |
| País Vasco | Guipúzcoa | Hospital Bidasoa | Jose Antonio Miguel Arce  Mª Asunción Celaya  Silvia Dorronsoro | Juan Antonio Miguel Arce |
| País Vasco | Vitoria | Hospital de Santiago Apóstol | Mª Inés Carrascosa | Mª Inés Carrascosa |
| País Vasco | Vizcaya | Hospital de Galdakao | Cristóbal Esteban  Mikel Egurrola  Alberto Capelastegui | Cristóbal Esteban  Mikel Egurrola |
| País Vasco | Vizcaya | Hospital de San Eloy | Jesús Camino  Juan Manuel Nuñez  Luis Alberto Ruiz Iturriaga | Jesús Camino |
| País Vasco | Vizcaya | Hospital Universitario de Cruces | Pilar Marín  José María Antoñana | Pilar Marín  José María Antoñana |
| País Vasco | Vizcaya | Hospital de Basurto | Miren Begoña Salinas  Igor Iturbe | Miren Begoña Salinas  Igor Iturbe |
| Valencia | Alicante | Hospital San Juan de Alicante | Eusebio Chiner Vives  Adaluz Andreu Rodríguez | Eusebio Chiner Vives  Cristina Senent Español  José Norberto Sancho Chust |
| Valencia | Alicante | Hospital de Orihuela | José Manuel Querol | Jose Manuel Querol |
| Valencia | Castellón | Hospital General de Castellón | Margarita Marín Royo | Margarita Marín Royo  German Llavador  Alfonso Martinez  Juliana Rissi  Maria Jose Bueso |
| Valencia | Valencia | Hospital General de Requena | Pablo Catalán Serra  Juan José Soler Cataluña | Pablo Catalán Serra |
| Valencia | Valencia | Hospital de Sagunto | Eva Martínez Moragón | Eva Martínez Moragón |
| Valencia | Valencia | Hospital Francisco de Borja | Concha Pellicer Ciscar | Concha Pellicer Ciscar |
| Valencia | Valencia | Hospital universitario de la Ribera | Elsa Naval Sendra | Elsa Naval Sendra |
| Valencia | Valencia | Hospital Clínico de Valencia | María Cruz González Villaescusa | María Cruz González Villaescusa  Paola Lisseth Ordoñez Gómez  Erick Leonardo Monclou Garzón  Maria Dolores Martínez Pitarch  Lucia Gil Maneu |
| Valencia | Valencia | Hospital Universitario Dr. Peset | Estrella Fernández Fabrellas  Ángela Cervera Juan  Alberto Herrerón Silvestre  Alfonso Martinez Martínez | Estrella Fernández Fabrellas  Ángela Cervera Juan  Anna Santabasilisa  Susana Herrera  Ruben Lera  Cristina Miralles  Belen Orosa |
